# Optimal quantum network decongestion strategies

**DOI:** 10.1038/s41598-023-36562-x

**Published:** 2023-06-17

**Authors:** Luca Perju Verzotti, Bogdan-Călin Ciobanu, Pantelimon George Popescu

**Affiliations:** Computer Science and Engineering Department, University POLITEHNICA of Bucharest, 60042 Bucharest, Romania

**Keywords:** Quantum information, Computer science

## Abstract

This study clarifies the problem of decongestion in quantum networks, with a specific focus on the crucial task of entanglement distribution. Entangled particles are a valuable resource in quantum networks, as they are used for most quantum protocols. As such, ensuring that nodes in quantum networks are supplied with entanglement efficiently is mandatory. Many times, parts of a quantum network are contested by multiple entanglement resupply processes and the distribution of entanglement becomes a challenge. The most common network intersection topology, the star-shape and it’s various generalizations, are analyzed, and effective decongestion strategies, in order to achieve optimal entanglement distribution, are proposed. The analysis is comprehensive and relies on rigorous mathematical calculations which aids in selecting the most appropriate strategy for different scenarios optimally.

## Introduction

Entanglement^1–3^ is a peculiar property exhibited by particles and systems of particles, that has no classical counterpart. Entangled pairs (e-pairs) are vital to quantum communications^4,5^ and enable distributed quantum computing^6–10^, quantum teleportation^11–13^, quantum key distribution (QKD)^14,15^, and others. One of the most important aspects of quantum communications is that entanglement fidelity^16^, due to its nature, is preserved only if the system does not have any interaction with the environment. As such, to be able to successfully transport entangled particles over long distances with high fidelity, the system should interact as little as possible with the environment while maintaining the ability to be manipulated and measured. Therefore, a great effort is put towards avoiding the physical transportation of e-pairs.

Photons are currently the most commonly used entangled particles in quantum networks^17–21^, as the matters of generating entangled pairs of photons, manipulating them, and measuring have been intensively studied. There are two mediums through which photons can be reliably transported: free space optical (FSO) and optical fiber. Interactions with the medium, such as the light absorption in optical fiber, lead to the loss of entanglement fidelity. Through free space optical intra-atmospheric transmissions, experimental qubits have been successfully transported to up to tens of kilometers^22^, while through optical fiber channels, a distance of 830km between nodes has been proven possible^23^. These limitations come in part from the fact that quantum signals cannot be amplified, as doing so would disturb the quantum state, leading to a loss of entanglement fidelity.

Due to the fact that fundamental procedures, such as teleportation and others, consume entanglement pairs during their processes, e-pairs can be viewed as a consumable in quantum networks. As such, a scheme to resupply entanglement between nodes in a network, needs to be put in place. Conceptually, the simplest approach would be to physically transport the pair towards the two requesting destinations, one particle for each. This approach has a high loss of entanglement fidelity, due to the fact that the entangled particles have to travel, at best, half the distance between the nodes. The transmission mediums through which the particles travel are lossy^22,24,25^, and aren’t perfectly isolated from environmental factors, thus, by travelling a longer distance, the fragile quantum states are exposed to a greater amount of interference. An improved approach can be achieved using the entanglement swapping and entanglement purification procedures^26–29^. Using quantum routing algorithms^30–34^, a route between two nodes requesting entanglement can be found, and by making use of entanglement swapping, segments of a route can be effectively traversed in parallel, leading to a decrease in the total distance a particle has to travel, as well as lowering the total transmission time.

The objective of this work is to propose and analyze the optimal strategy of handling simultaneous entanglement resupply requests, that have to contend over nodes and links within the same network. The paper is structured as follows. Through in "Overview of concurrent entanglement distribution challenges" section the setup of entanglement distribution networks is explored. In "Analysis of two intersecting simple entanglement distribution routes" section the scenario of a simple two-spoke star-shaped network is analyzed, and the two strategies are explained. In "Analysis of concurrency on large scale quantum networks" section presents more general star-shaped networks, along with an analysis for each of the considered scenarios regarding the optimal approach, in regards with the network conditions. The paper ends with conclusions regarding the optimality of the presented strategies.

## Overview of concurrent entanglement distribution challenges

**Figure 1:**
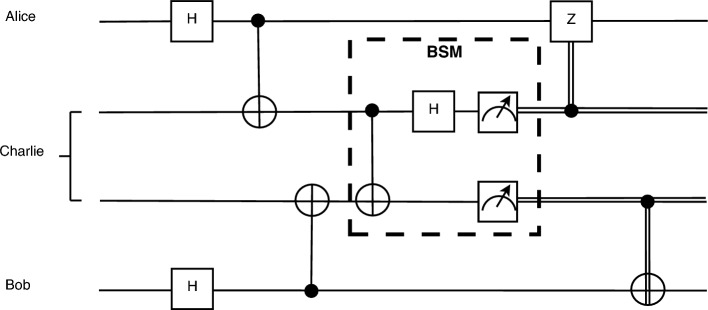
Entanglement swapping circuit.

We consider a homogeneous quantum network, where all nodes are quantum repeaters^35,36^ which can perform Bell State Measurements^37–39^ (BSM), included in the entanglement swapping protocol (see Fig. 1), and can produce e-pairs, without losing generality, i.e. if a node doesn’t have such capabilities, it will only forward the qubits, and therefore it will be considered as part of a link, not a standalone node. The first step, in order to construct an efficient distribution scheme, is to extract an optimal path between the two requesting nodes in the network. Then, the second step is to determine the nodes which will generate entanglement and how to optimally distribute it. Optimal protocols for selecting the path^40^ as well as optimal ways^41,42^ of performing the entanglement swaps on paths have been recently studied.

With the advent of increasingly large quantum networks, the problem of concurrency within these networks became important. Actual quantum networks, due to their smaller scale, do not exhibit resource contention often, as, for example, is the case for the DARPA Quantum Network^43^, where each node had a dedicated link toward it’s associated node.

For a global-scale Quantum Internet^32,44,45^, an efficient method to effectively decongest entanglement distribution networks would be critical. This stems from the fact that there is a multitude of protocols that require entangled pairs, and may consume them, in order to function (e.g. quantum teleportation, distributed quantum computing, quantum metrology, quantum clock synchronization. Having a lower throughput of e-pairs due to congestions in the network may lead to lower robustness of the protocols employed, as quantum error correction protocols can benefit from a higher throughput of e-pairs. Implicitly, the scalability of a global quantum internet is directly impacted by the efficiency of the entanglement distribution network that supplies the e-pairs to the end users.

The problem of concurrent entanglement distribution has been studied by Shi and Qian^46^, which proposes an algorithm that increases the throughput of a quantum network by always picking the path that guarantees the highest distribution rate and using the remaining resources to create backup paths. Their work emphasises that in order to increase the throughput of a quantum network, one needs to increase the success rate of the transmission, while also increasing the transmission speed by using methods such as entanglement swapping.

Another protocol that aims to maximize throughput through a quantum network has been proposed by Zeng et al.^47^. In their work, they have transposed the problem as two linear programming problems. By recovering the integer solutions, they are able to achieve better expected time than previous algorithms, while retaining a small computational overhead for determining the routes.

Without losing generality, we consider a scenario where there is only one link between nodes, that can transmit only unidirectionally at a given time. We also assume there is a certain duration $\tau _t$ which models the transmission time throughout the network. To have a uniform transmission time, we can assume that $\tau _t = \max {\tau _i}, i=0..n$, where there are *n* distinct links, each with a transmission time of $\tau _i$. Since we make use of entanglement swapping, we can consider a $time\text { }slot\text {}\tau$ as the transmission time through one link and the time associated to a subsequent measurement $\tau _m$, thus, $time\text { } slot$ can be described by the following equation.

$$\begin{aligned} \tau = \tau _m + \tau _t. \end{aligned}$$

Furthermore, we consider that a qubit has a decoherence time equal to $\tau$, which is a reasonable assumption and therefore, the necessity of QRAM^48,49^ is not justified.

In order to qualitatively describe the distribution strategies, the probability *p* is considered for successful transmission that is an average cumulative probability of transmission throughout the network links, and consider *q* as the probability for successful measurements. Recent advancements in this regard have increased the theoretical success probability of BSM^50,51^, while the probability of a successful transmission is strongly correlated with the length of the link. Therefore, throughout the rest of this manuscript, we will consider *p* and *q* as arbitrary probabilities without making any further assumptions.

**Figure 2:**
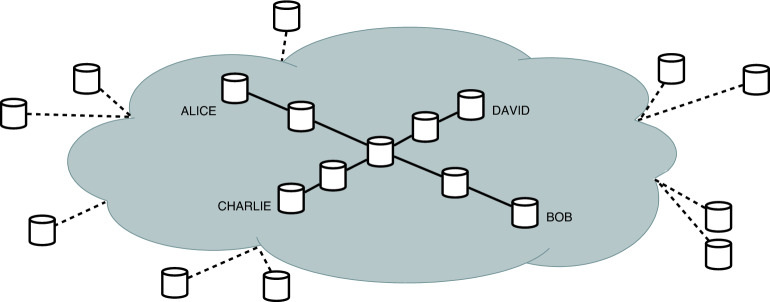
Network topology of concurrent entanglement distribution. Both Alice and Bob, and Charlie and Dave have requested e-pairs at the same time. Central node is the contested resource, as both networks need to pass an e-pair through it.

Throughout this work an analysis regarding how to decongest simultaneous entanglement resupply requests within the same network is presented. Due to the nature of quantum entanglement and the procedures such as entanglement swapping, we cannot serve requests simultaneously through the same node or link. We can however decongest the network through the proposed strategies. While the case where the routes do not intersect is trivial, for the case where there is concurrency for the nodes, we have identified two optimal approaches, described and compared in the following sections, determining under which conditions each of them should be used.

## Analysis of two intersecting simple entanglement distribution routes

Starting with the scenario where within one time slot, an entanglement request is served between two nodes: Alice and Bob (*A*, *B*), by using a chain of $l+1$ nodes (including Alice and Bob) connected with *l* quantum links. In order to minimize the expected time, we maximize the success probability for each time slot by performing the minimum amount of Bell measurements. All nodes situated on even positions (including Bob, when *l* is odd) should generate an e-pair and send one qubit in the direction of Alice and one in the direction of Bob. Afterwards, all nodes on odd positions, excluding Alice, will perform entanglement swapping, to establish long-distance entanglement. The success probability, in this scenario, is given by $P_{chain} = p^l*q^{\lfloor \frac{l-1}{2}\rfloor }$, where $p^l$ is the probability of all *l* transmissions working successfully, $\lfloor \frac{l-1}{2}\rfloor$ is the number of Bell measurements performed, and therefore $q^{\lfloor \frac{l-1}{2}\rfloor }$ is the probability of all those measurements being successful.

A more interesting situation appears when two simultaneous entanglement requests are received: (*A*, *B*) and Charlie-David (*C*, *D*), and their selected paths intersect, as in Fig. 2. We will first look at the case when both the paths from *A* to *B* and from *C* to *D* have a length of 5 nodes and are intersecting in the middle node, forming a star topology, with 4 spokes. We will first propose two strategies to solve this scenario, by a symmetrical approach, and then by an asymmetrical approach, and then analyze when each one should be used.

### Symmetrical approach

**Figure 3:**
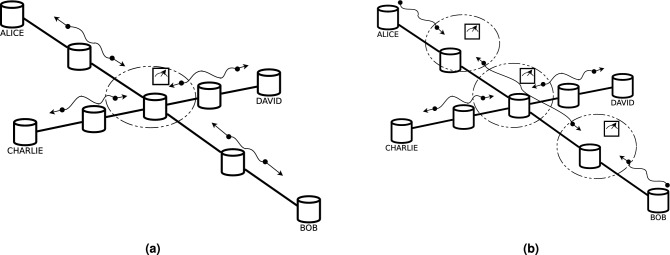
Network topology with the two possible distribution approaches; (**a**) represents the symmetrical approach, where on both routes the entanglement is generated and measured at the same node indices; (**b**) represents the asymmetrical approach.

In this distribution schema, see Fig. 3a, the second and fourth nodes from each route should each generate an e-pair, send one qubit toward the center, and the other one towards the end of the route. We will call this entanglement generation schema the *Even Generation*, as only the even numbered nodes generate e-pairs. The center node can now choose on which two qubits to perform entanglement swapping, resolving either the (*A*, *B*) or the (*C*, *D*) request but is unable to resolve both requests at the same time. This, however, is only an issue when all 8 transmissions were successful, since in every other case the center node will choose to perform entanglement swapping on the qubits which were successfully transferred. $\mathcalligra{T}^{sym}$, the expected number of time slots required to satisfy both requests by using this approach, can be calculated from

$$\begin{aligned} \mathcalligra{T}^{sym} = ( 1 - p^4 )^2 \mathcalligra{T}^{sym} + 2 p^4(1 - p^4) ( (1 - q) \mathcalligra{T}^{sym} + q \frac{1}{p^4q} ) + p^8 ( (1 - q) \mathcalligra{T}^{sym} + q \frac{1}{p^4q} ) + 1. \end{aligned}$$

### Asymmetrical approach

On one of the route, the second and fourth nodes generate an entangled pair, while on the other route the first, third, and fifth nodes do so (see Fig. 3b). In other words, a route uses *Even Generation*, while the other uses *Odd Generation* of entanglement. All those nodes then send one qubit to each of their neighbouring nodes on their respective routes.

For the route on which nodes 2 and 4 generated qubits, only the center node has to perform entanglement swapping, and for the other route, the second and fourth nodes will have to perform it. This way, if all transmissions and Bell measurements are successful, it is possible for both the (*A*, *B*) pair and the (*C*, *D*) pair to receive long-distance entanglement at the same time, in only one time slot. In realistic scenarios, where we have non-unitary probabilities for each one of the operations, we calculate the expected number of time slots required for this approach to resolve both pairs from

$$\begin{aligned} \mathcalligra{T}^{asym} = ( 1 - p^4q ) ( 1 - p^4q^2 ) \mathcalligra{T}^{asym} + p^4q ( 1 - p^4q^2 ) \frac{1}{p^4q} + p^4q^2 ( 1 - p^4q ) \frac{1}{p^4q} + 1. \end{aligned}$$

After calculating the expected performance of both strategies, we obtain

1 $$\begin{aligned} \mathcalligra{T}^{sym}&= \frac{p^4 - 3}{(p^4 - 2)p^4q}, \end{aligned}$$

2 $$\begin{aligned} \mathcalligra{T}^{asym}&= \frac{2p^4q^2 - q- 2}{(p^4q^2 - q - 1)p^4q}. \end{aligned}$$

**Figure 4:**
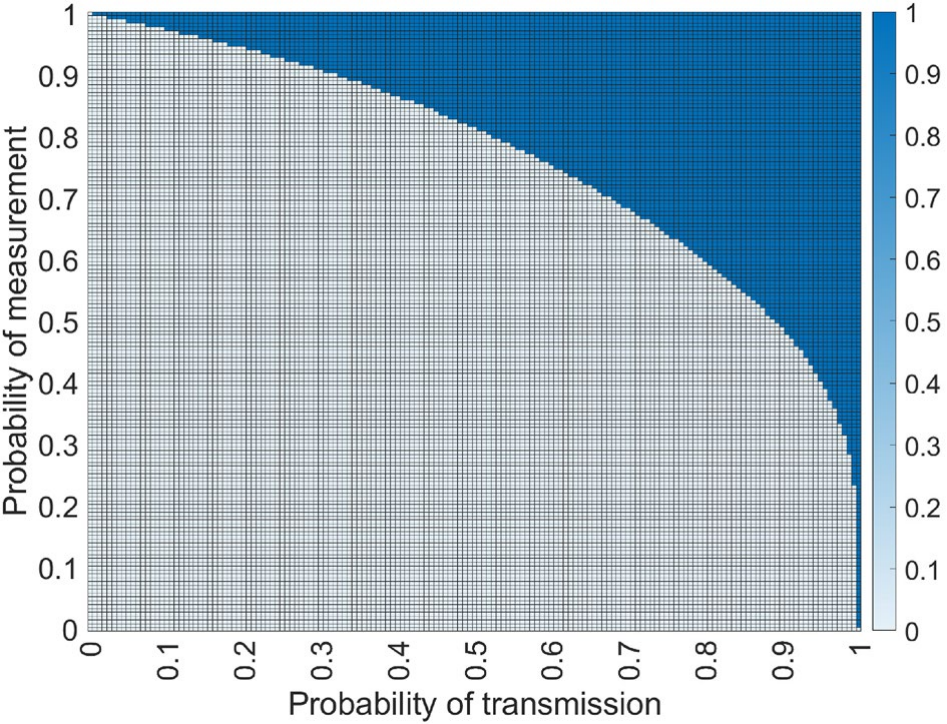
Symmetrical approach versus asymmetrical approach of two intersecting simple entanglement distribution routes. The white area represents the *p* and *q* values for which the symmetrical scheme yields a better expected time ($\mathcalligra{T}^{sym} \le \mathcalligra{T}^{asym}$) and with blue we have a better expected time using the asymmetrical scheme ($\mathcalligra{T}^{sym} > \mathcalligra{T}^{asym}$), considering Eqs. (1), (2).

Using these values we can compute the optimal strategy based on the probabilities *p* and *q* and, as expected, we can see from Fig. 4 that the asymmetrical approach performs better when the probabilities are high. This stems from the fact that it offers the possibility for both requests to be solved simultaneously, while the symmetrical approach is better for lower probabilities because it’s at a disadvantage only when all 8 transmissions are successful.

## Analysis of concurrency on large scale quantum networks

In this section we generalize the previously discussed network topology proposing new optimal entanglement distribution strategies. We want to emphasise that we consider the star-shaped network without loss of generality, as a subgraph resulting from intersecting routes between nodes in a graph, no matter how complex, would yield a generalized star topology. This generalized star topology would either have longer spokes, a greater number of spokes, have more than one node as the intersection between the spokes, or any combination of these. In this section we will analyse the effect of the first two of the variants separately, and the case where the intersection is a chain of multiple nodes. We will not consider any backup routes between the pairs.

### Analysis of two intersecting variable length entanglement distribution routes

Here we will assume that the paths (*A*, *B*) and (*C*, *D*) can have any variable lengths (*VL*), not necessarily equal, and the intersection, comprised of a single node, can be any node other than *A*,*B*,*C* or *D*. Firstly, we notice that if at least one of the paths has an odd number of edges then we will be able to independently solve each path using the optimal path protocol mentioned at the beginning of "Analysis of two intersecting simple entanglement distribution routes" section, since the nodes that generate e-pairs for the path with odd length can always be chosen such that they don’t interfere with the protocol for the other path. This also applies when both paths have an even number of edges when the intersection node is on an even position for at least one of the paths. In this case, the center node will need to generate one entangled pair and perform one BSM if it is situated on an odd position on the other path, or generate two entangled pairs without the need of performing any BSM when it’s on an even position for both paths. In the only remaining case, when both routes have an even number of edges, and the intersection node is located on odd positions for both paths, the reason why we can’t solve the paths independently is the fact that the center node is unable to perform two simultaneous BSM within the same timeslot. Thus, we will propose two possible optimal protocols.

#### Symmetrical approach

We will assume that the two lengths (in number of edges) are $l_1$ and $l_2$, with $l_1 \le l_2$. If all transmissions are successful on both paths, the intersection node should choose to perform entanglement swapping on the qubits belonging to the longer path, the one with length $l_2$. The expected number of required time slots required for the symmetrical approach to satisfy both entanglement requests regardless of the lengths of the paths will then be given by

$$\begin{aligned} \begin{aligned} \mathcalligra{T}^{sym}_{VL}&= ( 1 - p^{l_1} ) ( 1 - p^{l_2} ) \mathcalligra{T}^{sym}_{VL} + p^{l_1} (1 - p^{l_2}) \left( \left( 1 - q^{\lfloor \frac{l_1-1}{2}\rfloor } \right) \mathcalligra{T}^{sym}_{VL} + \frac{q^{\lfloor \frac{l_1-1}{2}\rfloor }}{p^{l_2}q^{\lfloor \frac{l_2-1}{2}\rfloor }} \right) \\ {}&+ p^{l_2} (1 - p^{l_1}) \left( \left( 1 - q^{\lfloor \frac{l_2-1}{2}\rfloor } \right) \mathcalligra{T}^{sym}_{VL} + \frac{q^{\lfloor \frac{l_2-1}{2}\rfloor }}{p^{l_1}q^{\lfloor \frac{l_1-1}{2}\rfloor }} \right) + p^{l_1+l_2} \left( \left( 1 - q^{\lfloor \frac{l_2-1}{2}\rfloor } \right) \mathcalligra{T}^{sym}_{VL} + \frac{q^{\lfloor \frac{l_2-1}{2}\rfloor }}{p^{l_1}q^{\lfloor \frac{l_1-1}{2}\rfloor }} \right) + 1. \end{aligned} \end{aligned}$$

Since this distribution scheme is only used when both length are even, in order to simplify the equation we will assume that both $l_1$ and $l_2$ are even. Therefore, by calculus we obtain

3 $$\begin{aligned} \mathcalligra{T}^{sym}_{VL} = \frac{p^{2l_1+l_2}q^{l_1} - p^{l_1+l_2}q^{l_1/2+l_2/2} - p^{2l_1}q^{l_1} - p^{2l_2}q^{l_2}}{\left( p^{l_1+l_2}(q^{l_1/2}+q^{l_2/2} - q^2) - p^{l_1}q^{l_1/2} - p^{l_2}q^{l_2/2}\right) p^{l_1+l_2}q^{l_1/2+l_2/2-1}}. \end{aligned}$$

**Figure 5:**
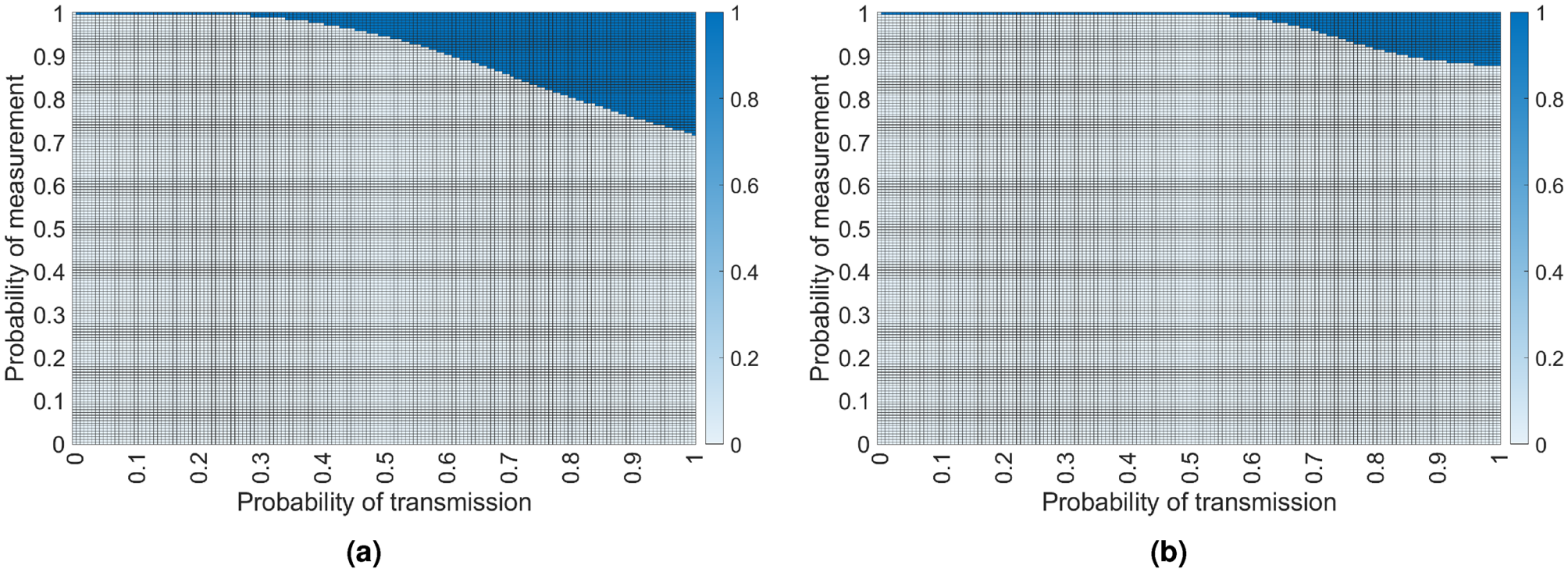
Symmetrical approach versus asymmetrical approach of two intersecting variable length entanglement distribution routes. The area in white shows under which circumstances the symmetrical scheme yields a better expected time $(\mathcalligra{T}^{sym}_{VL} \le \mathcalligra{T}^{asym}_{VL})$, while blue corresponds to a better time using the asymmetrical scheme $(\mathcalligra{T}^{sym}_{VL} > \mathcalligra{T}^{asym}_{VL})$, considering Eqs. (3, 4). In (**a**), we have used $l1 = 4$, $l2=6$, while for (**b**) $l1=10$, $l2=10$.

#### Asymmetrical approach

Let the two lengths (in number of edges) be $l_1$ and $l_2$. We will use a variation of the Asymmetrical approach where we have to choose which of the two paths will perform the extra BSM. It turns out it is always optimal for the shorter path to perform the extra BSM. If we assume that $l_1 \le l_2$, we will obtain the expected number of time slots required to entangle both requesting pairs from

$$\begin{aligned} \begin{aligned} \mathcalligra{T}^{asym}_{VL}&= \left( 1 - p^{l_1}q^{\lfloor \frac{l_1+1}{2}\rfloor } \right) \left( 1 - p^{l_2}q^{\lfloor \frac{l_2-1}{2}\rfloor } \right) \mathcalligra{T}^{asym}_{VL} + p^{l_1}q^{\lfloor \frac{l_1+1}{2}\rfloor } \left( 1 - p^{l_2}q^{\lfloor \frac{l_2-1}{2}\rfloor }\right) \frac{1}{p^{l_2}q^{\lfloor \frac{l_2-1}{2}\rfloor }} \\&+ p^{l_2}q^{\lfloor \frac{l_2-1}{2}\rfloor } \left( 1 - p^{l_1}q^{\lfloor \frac{l_1+1}{2}\rfloor }\right) \frac{1}{p^{l_1}q^{\lfloor \frac{l_1-1}{2}\rfloor }} + 1. \end{aligned} \end{aligned}$$

By assuming that $l_1$ and $l_2$ are even, we obtain

4 $$\begin{aligned} \mathcalligra{T}^{asym}_{VL} = \frac{p^{l_1+l_2}q^{l_1/2+l_2/2} + p^{2l_1}q^{l_1+1} + p^{2l_2}q^{l_2} - p^{l_1+2l_2}q^{l_1/2+l_2} - p^{2l_1+l_2}q^{l_1+l_2/2}}{\left( p^{l_1}q^{l_1/2+1} + p^{l_2}q^{l_2/2} - p^{l_1+l_2}q^{l_1/2+l_2/2}\right) p^{l_1+l_2}q^{l_1/2+l_2/2-1}}. \end{aligned}$$

It can be observed from Fig. 5 that the conditions under which the asymmetrical approach would yield a faster resupply time are becoming stricter as the length of the spokes increases. This can be explained by the fact that, as the length of the spokes increases, the probability that all transmissions and measurements are successful decreases. As such, it would be better to hedge the two requests, in the probable eventuality that one of them will fail.

### Analysis of many intersecting simple entanglement distribution routes

**Figure 6:**
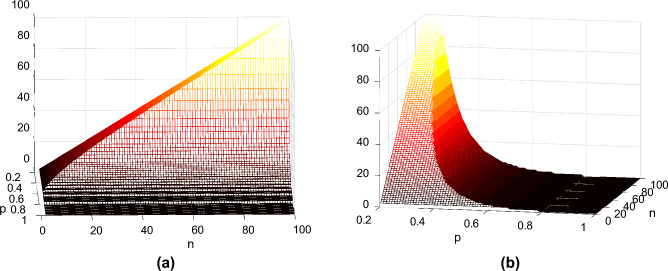
Optimal number of $Even\text { } Distributions$ in the context of many intersecting routes, considering $q=0.625$, seen from two perspectives (**a**) and (**b**).

In this scenario, we assume that there are more than 2 pairs requesting entanglement, with only one node as the intersection between their respective routes, and, for simplicity of the equations, we consider the lengths of the spokes to be 2. We will consider again two possible generation schemas - even and odd. Since the center node can generate as many e-pairs as needed at the beginning of every distribution attempt, each path using the odd schema will have an independent probability of success of $p_o = p^4q^2$. Among the paths using the even schema, however, only one will be able to be served, as the intersection node isn’t able to perform more than one BSM per timeslot. Therefore, we obtain that when *i* paths use the even schema, the probability of at least one of them succeeding is equal to $p_e^i = (1 - (1 - p^4)^i)q$. What remains is finding how many pairs should use each of the two schemas for the optimal distribution. For this, we start by calculating $t_n$, the expected number of timeslots required to serve *n* requests for some fixed probabilities *p* and *q*.

The values of $t_n$ can be calculated recurrently by choosing the optimal number of paths to use the even schema, among all posibilities, as following

$$\begin{aligned} t_0= & {} 0, \\ t_1= & {} t_0p_e^1 + t_1(1-p_e^1), \\ t_2= & {} \min \{t_0p_e^1p_o + t_1p_e^1(1-p_o) + t_1(1-p_e^1)p_o + t_2(1-p_e^1)(1-p_o), t_1p_e^2 + t_2(1 - p_e^2)\}, \\ {}&\cdot \cdot \cdot&\\ t_n= & {} \min _{\forall i \in [1,n]} \left\{ \sum _{j=0}^{n-i+1} t_{n-j}((p_o)^j(1-p_o)^{n-i-j} \left( {\begin{array}{c}n-i\\ j\end{array}}\right) (1-p_e^i) + (p_o)^{j-1}(1-p_o)^{n-i-j+1} \left( {\begin{array}{c}n-i\\ j-1\end{array}}\right) p_e^i \right\} . \end{aligned}$$

In the above formula, *i* represents the number of paths using the even generation schema. Therefore, the goal is finding the *i* that results in the smallest expected number of time slots required, $t_n$. We observe that regardless of the probabilities *p* and *q*, the value of *i* that minimizes the expected time is always equal to the one that maximises the expected throughput for every single timeslot. By computing $\mu _n^i$, the expected number of pairs served within a timeslot out of *n* requesting pairs while using the even schema for *i* of the paths, and the odd schema for the remaining $n-i$, we get

$$\begin{aligned} \mu _n^i = p_e^i + (n-i)p_o. \end{aligned}$$

By substituting $p_e^i$ and $p_o$ and maximising $\mu _n^i$ in the above equation we will obtain $i_{opt}$, the number of paths that should use the even generation schema, based on the values of *p*,*q* and *n*

$$\begin{aligned} {i_{opt}} = \min \left\{ n, \Big \lceil {\frac{\ln {q}}{\ln {(1-p^4})}}\Big \rceil \right\} \end{aligned}$$

In Fig. 6 we plot $i_{opt}$, the maximum number of even schemas that should be used, based on the values of *p* and *n* by assuming that $q=0.625$ (a common value for BSM). The most poignant feature of these graphs is that for bad network conditions ($p, q < 0.4$), the optimal number of even spokes tends to approach the number of total spokes. For $p\approx 0.3$, $i_{opt}$ is in fact equal to *n*. In Fig. 6b, the relatively few values that are associated for high *p* and *q* values can be observed through the visible steps that the surface has. For relatively better network conditions, the optimal number of even schemas remains relatively low. Intuitively, this means that, if the chances of successful distribution are low for both schemas, it is better to focus resources on getting at least one e-pair transmitted, through the most reliable method of the two.

In Algorithm 1 we outline the step-by-step process for selecting the optimal method for distributing entanglement in a multiple request scenario, as discussed in this section. This approach takes into account the specific parameters of the intersection, providing a sysematic way to determine the best course of action.
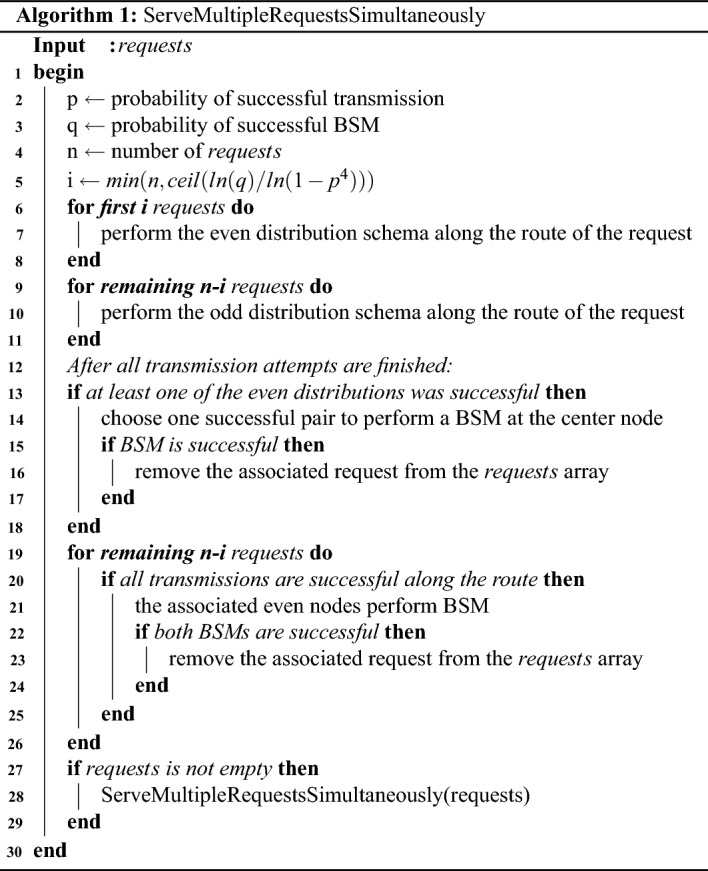


### Analysis of intersecting chain of two entanglement distribution routes

**Figure 7:**
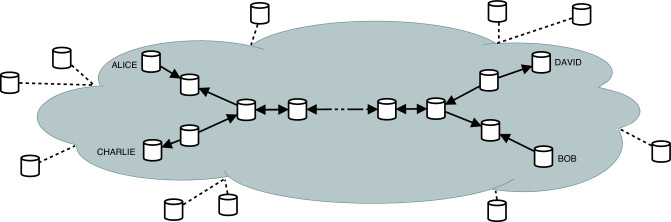
Intersecting chain of two entanglement distribution routes.

In the case of multiple nodes in a chain topology within the actual intersection, due to the restrictions that we have imposed on the network, namely that a link can only transmit in one direction within a timeslot, asymmetrical approach is not possible. This occurs due to the fact that in asymmetrical approach, one e-pair is inbound to the central node, while another is outbound. If we were to expand that central node to a chain, as shown in Fig. 7, any outbound or inbound pairs would have to travel between the ends of the chain. This would imply that between all the consecutive nodes in the chain, two qubits are exchanged, which is impossible.

Therefore, within the constraints that we have imposed, the only viable strategy is to perform the symmetrical scheme, which would have the greatest chance of success, but could only be serving one requesting pair per timeslot. One way to enable the asymmetrical scheme would be to have bidirectional links between the nodes, in which case the chain would only act as an extension of the spokes, and the results from "Analysis of two intersecting variable length entanglement distribution routes" section would apply.

## Conclusion

In this paper we have presented an approach to a previously unexplored problem, namely optimal handling of congestion in a quantum network. We have proposed two strategies to solving congestion due to intersecting routes in a quantum network: symmetrical and asymmetrical schemes. In a basic example of two intersecting routes, a symmetrical approach implies that neither route requires knowledge of one another, and both execute a generation scheme where e-pairs are generated in even numbered nodes, and distributed in both directions. An asymmetrical scheme implies that one route employs an even generation, while the other an odd generation.

Both approaches have their merits, and, as we have shown, their usecases. The symmetrical approach works best when the probability of transmission is fairly low through the optical links of the network. Even though only one of the requesting pairs can be served within a time slot, this way, we can maximize the chance that at least one of the pairs gets entanglement. Thus, the symmetrical method is the most resilient of the two approaches to low quality transmissions through the network.

The main advantage of the asymmetrical schema is that it has the potential to simultaneously serve two pairs within a single time slot. However, due to the nature of odd generation of entanglement, this results in one more BSM than the symmetrical variant, leading to a lower chance of overall success. This distribution schema would be preferred to maximize throughput in networks with high transmission and measurement probabilities.

We have analyzed the effectiveness of these strategies through more generalized intersections, such as star shaped topologies with long spokes, more than 4 spokes, and more nodes at the intersection. We have found that the asymmetrical schema is still viable with longer spokes, albeit with smaller tolerances for successful measurement and transmision. We have also described the equation that gives the optimal distribution of even and odd generations for more numerous requesting pairs, such that we can serve the most number of pairs in the least amount of time slots. Finally, we have concluded that with more nodes at the intersection, in a chain, we cannot perform an asymmetrical distribution, thus we can only deliver a maximum of one e-pair per timeslot.

As future work, we propose an analysis of strategies over an even more general star-shaped network topology, where all the 3 subcases are compounded. An analysis of different types of topology like crossbars and other interconnection networks, and the effect of backup routes upon these distribution schemas.

## Data Availability

The datasets generated during the current study and the complete mathematical calculus will be made available from the corresponding author on reasonable request.
